# Gas Plasma Technology—An Asset to Healthcare During Viral Pandemics Such as the COVID-19 Crisis?

**DOI:** 10.1109/TRPMS.2020.3002658

**Published:** 2020-06-16

**Authors:** Sander Bekeschus, Axel Kramer, Elisabetta Suffredini, Thomas von Woedtke, Vittorio Colombo

**Affiliations:** 1 ZIK plasmatis, Leibniz Institute for Plasma Science and Technology (INP)17489GreifswaldGermany; 2 Leibniz Networks on Health Technologies and Immune-mediated Diseases; 3 Institute for Hygiene and Environmental Medicine, Greifswald University Medical Center17489GreifswaldGermany; 4 Department of Food Safety, Nutrition and Veterinary Public HealthIstituto Superiore di Sanità 9289 00161RomeItaly; 5 Leibniz Network on Health Technologies; 6 Department of Industrial Engineering, Interdepartmental Center for Agri-food Industrial Research, Interdepartmental Center for Industrial Research on Advanced Applications in Mechanical Engineering and Materials TechnologyAlma Mater Studiorum-Università di Bologna 9296 40136BolognaItaly

**Keywords:** Airborne virus, cold atmospheric pressure plasma, DBD, decontamination, plasma jet, transmission

## Abstract

The COVID-19 crisis profoundly disguised the vulnerability of human societies and healthcare systems in the situation of a pandemic. In many instances, it became evident that the quick and safe reduction of viral load and spread is the foremost principle in the successful management of such a pandemic. However, it became also clear that many of the established routines in healthcare are not always sufficient to cope with the increased demand for decontamination procedures of items, healthcare products, and even infected tissues. For the last 25 years, the use of gas plasma technology has sparked a tremendous amount of literature on its decontaminating properties, especially for heat-labile targets, such as polymers and tissues, where chemical decontamination often is not appropriate. However, while the majority of earlier work focused on bacteria, only relatively few reports are available on the inactivation of viruses. We here aim to provide a perspective for the general audience of the chances and opportunities of gas plasma technology for supporting healthcare during viral pandemics such as the COVID-19 crisis. This includes possible real-world plasma applications, appropriate laboratory viral test systems, and critical points on the technical and safety requirements of gas plasmas for virus inactivation.

## I. Introduction

The name of the event with the most dramatic impact on human societies and financial and healthcare systems across the globe in the 21st century so far is “COVID-19 pandemic.” Particularly, frightening about the pandemic is the speed of its worldwide spread due to the nature of the severe acute respiratory syndrome coronavirus 2 (SARS-CoV-2) virus being transmitted in aerosol droplets released during the speaking, breathing, and coughing of infected people. The pandemic ruthlessly dismantled some of the shortcomings in hospitals, disease diagnostics, and public life when it comes to dealing with extremely infectious pathogens. At the same time, many governments have also revealed unpreceded flexibility and willingness to act upon the daily changing situations. It can be hoped that the year 2020 will be marked as the year in which the global community learned that a robust protection strategy is needed to combat health crisis like this, as it will be utterly needed in the event of other pandemic outbreaks, whose lethality, based on social and health context and depending on concurrent adverse events, might also exceed what we are currently experiencing with COVID-19, as demonstrated by the Spanish flu in the early 20th century.

The main pillar of fighting a pandemic is to break infection chains. This means disallowing infected people to spread the virus not only by containment but also by performing rigorous disinfection measures of contaminated goods and devices, decontamination of indoor air, especially in operation theaters with mixed ventilation as well as antiseptic prevention and treatment. While the mostly alcohol-based disinfection is suitable for the majority of applications, some cases would benefit from optimized decontamination procedures to help to limit virus spread. During the past months, it was natural to discuss with colleagues within and across scientific disciplines as well as societal stakeholders the options of gas plasma in helping to reduce viral loads wherever this technology might be advantageous over existing methods. We here outline some of the perspectives on how plasma might be useful during a pandemic like COVID-19 after giving a brief introduction on the topics related to this. The aim is to provide the information in a way to be comprehensible to scientists from several disciplines, including physicists, biologists, societal stakeholders, medical doctors, and healthcare practitioners. After all, history has shown that bringing together ideas from several fields often is the key to find new solutions, such as the need for alternative or complementary virus inactivation methods to upgrade existing procedures: a lesson tragically learned by recent viral pandemics, such as COVID-19.

## II. Viruses and COVID-19

### A. Viruses Highjack

The SARS-CoV-2, the etiological agent of the pandemic called Coronavirus disease 2019 (COVID-19), is a virus associated with infections mainly of the respiratory system as well as other organs and systems. In contrast to eukaryotic cells (such as cells of the human body) and prokaryotic cells (bacteria), viruses do not display a self-maintaining metabolism. This essentially means that eukaryotic and prokaryotic cells can live and multiple based on nutrients from the environment, while viruses are not able to replicate on their own. In contrast, they need a host cell to perform virus replication for them. The virus is highjacking the host cell for producing the proteins and nucleic acids necessary to build new virus particles. For some types of viruses, this also extends to lipids. Eventually, the virus-infected cells turn into virus-producing factories. In a chain reaction, the new virus particles can then enter adjacent cells and infect them, or they can be transported outside the body exploiting existing exit pathways (i.e., the respiratory system or the digestive/urinary tract).

### B. Viruses Mutate

From a biological point of view, it is vital to acknowledge that all living organisms on Earth have co-evolved with viruses. Virus infections are known in, e.g., bacteria (called phages), fungi, plants, and vertebrates. It is speculated that the DNA of humans and many other organisms contain many regions originally stemming from viral genomes of ancient times, exemplifying the long-standing interaction of living cells with “parasitic” DNA. Another vital aspect of viruses is their capability, in terms of their nucleic acid sequence, of evolving quickly, compared to eukaryotic organisms. During replication in the host cells, a more than a trillion-fold event in a COVID-19 patient [1], the nucleic acid sequence replication accumulates errors. Some of these errors are beneficial to the virus (e.g., by increasing its infection or latency rate), some are without consequences (neutral), and, finally, some are detrimental to the virus’ spread. The SARS coronavirus of the 2003 epidemic, the first to be transmitted by air travel [2], showed a moderate mutation rate [3], and a similar evolutionary rate is reported for SARS-CoV-2 [4]. Changes in the nucleic acid sequence are also used to track the spreading of COVID-19 during the 2020 pandemic. Nowadays, sequencing technologies are fast, accurate, and affordable, so that these mutations can be utilized to cluster different variants of the current pandemic coronavirus [5]. After all, it is essential to understand that from a virus’ perspective, a maximum spread at moderate lethality is preferable, as swiftly killing the host will eventually kill the virus, hence interrupting its chance for further spreading.

### C. Coronaviruses Are Enveloped RNA Viruses

Viruses can be classified according to several criteria. One is whether they are enveloped or nonenveloped. Another is whether the information necessary for viral replication is stored on DNA or RNA. Similar to influenza viruses, coronaviruses are enveloped RNA viruses. The envelope, essentially a part of the host cell membranes lined with viral proteins, is one factor determining the virucidal efficacy of disinfection [6], [7]. DNA and RNA viruses differ in many aspects. The genome of DNA viruses is stable, usually larger than in RNA viruses, and the replication is accurate. In contrast, the genome of RNA viruses is less stable, error prone in replication (due to the absence of proofreading activity in RNA-dependent polymerases codified in viral genomes), and usually codes for less content (small genomes). Some DNA viruses, as well as some RNA viruses, can integrate into the genome of the host cell and remain latent (“asleep”) until, e.g., cellular stress and viral replication occur. Well-known examples are herpesvirus, HIV, and papillomavirus. Because of this these viruses may permanently persist in the host throughout its lifetime. In contrast, some of the most epidemiologically relevant RNA viruses (which include, for example, the largest part of the viruses responsible for gastroenteritis, and enteric viruses such as, poliovirus and hepatitis $A$ and $E$ viruses) replicate in host cell cytosol and quickly spread virus particles to the environment at high concentrations, only rarely displaying a long-lasting persistence in the host. Prominent examples of RNA viruses are norovirus, Ebola, measles, and coronavirus. These aspects of virus diversity are important when evaluating the literature on virucidal studies. Similar to bacteria, the more apart the investigated species are, the poorer any translational of results are from one species to another. Readers further interested in virus taxonomy and intrataxon virus divergence are referred to as an updated 15-rank classification hierarchy of viruses published in May 2020 [8].

## III. Gas Plasma Technology and Decontamination

### A. Principles of Gas Plasma Technology

Physical plasma is an excited gas state, sometimes called “the fourth state of matter,” that can be generated by a continuous supply of energy to the atoms or molecules of a neutral gas. Even if the energy required may be provided separately by thermal, chemical, electrical, and radiative resources or by a combination of all, the predominant ionizing mechanism is the collision process that involves an inelastic collision, electron impact, radiative interactions, and charge exchange. The typical life span of excited states is about 10 ns, i.e., if the energy supply is stopped, a depletion process starts, rapidly quenching the plasma. The most robust procedure of generating a plasma for biomedical purposes is electron impact ionization based on the application of a strong electric field. The electron energy is transferred by inelastic and elastic collisions with the atoms or molecules in the gas, resulting in its full or partial ionization. The temperature of partially ionized gas is always substantially lower than the characteristic ionization temperature. Dependent on the design of a plasma source, an ambient temperature of the plasma can be achieved, making it very interesting for the treatment of heat-sensitive surfaces and materials. The complex physicochemical plasma characteristics depend on a multitude of parameters, including the type and composition of the gas or gas mixture used for plasma generation, the applied energy and electrode configuration, the pressure, and the environment. With regard to its application, especially in the medical context, useful classifications are thermal versus nonthermal plasmas and low pressure versus atmospheric pressure plasmas [9]–[11].

In the last few years of the steadily growing development of plasma medicine as a new field of disciplines, four cold atmospheric plasma sources have undergone medical clinical trials in Germany and were licensed as medical devices. They operate either as direct dielectric barrier discharge (DBD) or indirect surface DBD or as plasma jet [12]–[14]. The benefit of these sources is that they can be operated at atmospheric pressure in ambient (“room”) air, possess temperature similar to that of the human body, are stable and easy to operate, and are relatively cost effective in terms of manufacturing.

### B. Survival of SARS-CoV-2 in the Environment

The SARS-CoV-2 virus is primarily released from the respiratory tract as an aerosol, which can be increasingly released through intubation, bronchoscopy, rhinoscopy, or surgical interventions, for example, [15] and [16]. SARS-CoV-2 is also detectable in the blood, with a prevalence of 15%, which can contaminate the surgical team during interventions [17]. The virus is present in 25%–80% of stool samples as well, which may lead to environmental and surface contamination in hospital and household settings, with the need for accurate surface disinfection [18], [19]. SARS-CoV-2 can survive in indoor air as an aerosol for 3 h. Noninfectious virus particles were detectable on copper surfaces after 4 h and cardboard after 24 h [20]. After two and three days, respectively, viable viruses still could be detected on stainless steel and plastic carriers. In similar studies with higher virus titers of SARS-CoV-1, the viruses remained intact up to six days [21]. Therefore, methods for killing respiratory viruses as SARS-CoV-2 in indoor air as well as on surfaces are required.

The use of gas plasma technology is successful or promising for hygienic indications when antimicrobial, antiviral, and/or antibiofilm activity is required. This is especially relevant for materials that are thermolabile or sensitive against chemical microbicidal active agents and/or when microbicidal chemical agents cannot or only insufficiently reach the site of action. Because of the highly effective antimicrobial components of gas plasma, the spectrum of its activity includes all vegetative bacteria species, including multidrug-resistant organisms as well as a broad spectrum of viruses [22] and even prions known for their extraordinary resistance. An intrinsic resistance against gas plasma treatment has not been observed, given its mode of action [23]. Respiratory viruses, such as avian influenza virus and Newcastle disease virus, were inactivated on surfaces using gas plasma within 2 min of treatment [24] and within 5 min of treatment for the human respiratory syncytial virus (RSV) [25]. An unsolved problem is the decontamination of indoor air in operation theater with mixed ventilation, especially during orthopedic and traumatic interventions, because a mixed infectious aerosol from the respiratory tract and blood dust endangers the surgical team. The ventilation system can increase the exposition of the team to SARS-CoV-2. Only if laminar airflow with direct expulsion of exhaust air from the operating room to the outside is present, and if the ventilation system can be switched to negative pressure, the risk of infection is minimal. Operating rooms with turbulent mechanical ventilation or without ventilation are not acceptable for orthopedic and traumatic surgery on COVID-19 patients. A safe technical solution is an innovative mobile filter system, which first sucks in room air through an M5 prefilter [26], [27]. Then, the air passes through a G4 carbon pleated filter. Inside the unit, the sucked-in air is decontaminated from microorganisms, including viruses using a gas plasma field and, at the same time, detoxified (e.g., allergens and odor pollution). The purified air is then fed back into the room through a HEPA 13 filter, which completely retains microorganisms, and then through a G4 pleated carbon filter from the top side of the unit (currently, ongoing clinical trial NCT02695368). In a proof-of-concept study using MS2 bacteriophages as a surrogate for influenza virus, 99.99% (4 logs) of the virus particles were inactivated within 15 min after nebulization in indoor air (unpublished observation of Balarashti and Conley from the Aerosol Research and Engineering Laboratories). The ozone concentrations released from the device were below the recommended limit. The idea that gas plasmas are active against MS2 bacteriophages was underlined in two other studies, where a successful inactivation using gas plasma technology after only 0.12 s [28] and 0.25 s [29] of contact time of the aerosol with the gas plasma occurred.

### C. Gas Plasmas as Universal Tool for Decontamination

The application of gas plasma to inactivate, kill, or remove pathogens is under research for more than 50 years. Initially, much work has been done on low-pressure plasmas for antimicrobial treatment of materials and devices. The enhanced availability of atmospheric pressure gas plasma devices from the middle of the 1990s led to intensified investigations for their use for antimicrobial treatments. Even if for these efforts in many cases, the term “plasma sterilization” was claimed, a real plasma-based sterilization process or device that meets the requirements of sterility assurance is not available yet [30]. Even if, because of several practical and regulatory reasons, gas plasma processes might be not suitable to replace or equally complement classical sterilization processes, there is an enormous potential to use gas plasma processes for specific decontamination purposes in hygiene and medicine where conventional measures based on heat, radiation, or toxic chemicals are not effective or not applicable [31]–[38].

Large-scale plasma devices harbor enormous economic and healthcare potential. The COVID-19 outbreak has led to dangerous shortages of sterilization capacity at hospitals. Plasma-supported hydrogen peroxide gas sterilization using gas plasma technology is an effective alternative to conventional and/or traditional sterilization methods and an indispensable innovation for the sterilization of polymeric and heat-sensitive medical devices [39]–[41]. In food processing, extensive research activities already brought in some cases very high technology transfer level (TRL) [42]. This relates to treating foods, studying the effects of plasma treatment on food constituents and qualities in different matrices, as well as in food safety and in packaging techniques to increase the shelf life of products. In the field of water cleaning, the capabilities of nonthermal plasmas in contact with liquids as an efficient oxidative degradation means have also been investigated due to the fact that conventional treatments are unable to remove nonbiodegradable pharmaceutical compounds [38], [43].

Another challenging but promising application is antisepsis using gas plasma technology. Gas plasma tolerability has been shown for skin [44]–[46] and wounds [47] and can be assumed analogously for mucosal tissue. As vaccination against COVID-19 is not available yet, all possible hygienic preventive measures must be exhausted in order to protect especially medical staff. Maximum efficacy and safety provided, the use of plasma devices may also be an addition or alternative to alcoholic hand disinfection. Gas plasmas were highly effective in eradicating physiological and artificial microorganisms on the fingertips of healthy volunteers [48]. Gas plasma treatment was well tolerated, and neither damaged the skin barrier nor caused skin dryness [49]. However, for gas plasma-based hand disinfection to be practical, plasma exposure times would need to be reduced significantly by technical means.

### D. Gas Plasma Treatment of Viruses

In the frame of gas plasma treatment playing a role in the reduction and control of airborne virus transmission through droplets and to reduce the burden of disease and control the nosocomial spread of respiratory viruses in the hospital setting, the work of Terrier *et al.* [50] has to be taken into account. High titer suspensions of influenza virus type $A$, human parainfluenza virus type 3, and RSV have been subjected to treatment by a combination of cold oxygen plasma and UV light. The system consisted of a one-pass flow tunnel where contaminated droplets of diameters in the micrometer range were nebulized and then treated with an internal gas device (plasma combined with UV light), with air being sampled before and after the reaction in the device. Virus inactivation efficiency in various operating conditions for the system was tested, and an up to 6.8 log decrease was reported. For adenoviruses inactivation in liquids, Zimmermann *et al.* [51] used a flat surface dielectric barrier microdischarge technology operating in air. Here, a very strong virus inactivation was observed as well, being up to 6 logs following a 4-min gas plasma exposure. For herpes simplex virus type 1 (HSV-1) strain 16, Brun *et al.* [52] used a plasma source based on the ionization of helium flow. However, a reduction in virus infectivity was not observed after plasma treatment. Another study used plasma-treated cell culture medium and exposed HSV-1 *in vitro*, on human corneal epithelial cells, and *ex vivo*, on explanted corneas, without identifying pronounced toxicity. At the same time, a marked antiviral activity was observed. The results demonstrated a good chance of translating plasma to the clinic for use against drug-resistant herpes keratitis [53]. Another type of virus persistently troubling patients and the medical field is the hepatitis $B$ virus (HBV), especially in transfusion medicines responsible for generating blood supplies for patients. Using a direct DBD, blood containing HBV antigen was exposed to gas plasma. The plasma treatment decreased the antigenicity of the particles, while significantly disturbing red blood cell homeostasis and inducing hemolysis. It can be concluded that gas plasma, at least for DBDs at the given setting and with whole blood, is not suitable to free blood samples from virus contaminations [54]. HIV is also an issue for transfusion medicines. Using a different model than blood, Volotskova *et al.* investigated the effects of treatment with a helium plasma jet on HIV-1 replication in monocyte-derived macrophages (MDMs). The anti-HIV activity of plasma treatment was observed, inhibiting effect on virus–cell fusion, viral reverse transcription and integration, and virus particles produced by gas plasma-treated cells showing reduced infectivity [55]. In another study on HIV [56], Amiran *et al.* exposed HIV to a plasma of a helium jet. The results showed increased inhibition of HIV with increasing voltage and plasma treatment time that, however, was already too toxic for the HeLa cells used as an infection model, discouraging this approach in the particular model used. All studies mentioned above were done at atmospheric pressure. At nonatmospheric pressure, a virucidal effect of nitrogen gas plasma on the influenza virus and its components was observed [57].

An easy way to test virucidal activity is through the use of bacteriophages. After a challenge, the infectivity of the phages can be assessed by their potency to lyse bacteria. Wu *et al.* investigated new strategies for the containment of airborne (and waterborne) transmission of viral agents. They studied the plasma-assisted inactivation of MS2 bacteriophages using a DBD plasma source set in an exposure chamber and injected with the aerosolized viruses from an MS2 suspension [28]. To test waterborne conditions, the same MS2 bacteriophage was plasma treated while being in suspension (not airborne). Inactivation of the MS2 viruses for both the airborne and waterborne states depended on the power level, exposure time, and carrier gas, and was primarily attributed to the plasma-generated reactive oxygen species that mediated oxidative damage to the virus surface proteins and RNA. Along similar lines, Guo *et al.* investigated the mechanism of virus inactivation (water containing surrogate virus-bacteriophages) by reactive oxygen and nitrogen species generated by a surface DBD plasma system. The treatment effectively damaged phage protein and RNA. Moreover, treatment with plasma-treated water was similarly effective than direct plasma treatment, suggesting long-lived reactive species—easily to be manufactured chemically—to be the primary agent of mediating inactivation [58]. Phages were also used as a model to demonstrate the antiviral activity of an industry-scale prototype aiming at containing the transmission of airborne infectious diseases [29]. Viral aerosols in airstreams (up to 330 L/min) were subjected to nonthermal gas plasma treatment within a packed-bed reactor where a DBD was found to inactivate aerosolized MS2 phages with increasing applied voltage. Particular care was also given to the downstream treatment of residual ozone using activated carbon filters. In general, a more extensive review of gas plasma studies on virus inactivation was recently published [59].

While most of these studies were rather descriptive in terms of mechanisms and redox chemistry that may be optimal or suboptimal for antiviral activity, the group of Bruggeman has published a sophisticated study, shedding more light on these aspects. A remote radio-frequency plasma source, feline caliciviruses, an array of feed gas compositions, and a dozen of different radical scavengers were used in this article [60]. In brief, they found two plasma-induced chemistries, each with distinctive main pathways of inactivation, either being based on singlet oxygen or peroxynitrous acid. In general, it has to be acknowledged that the mechanisms on gas plasma-assisted virus inactivation are not fully understood. It can be assumed that the virus particles are subjected to destructive oxidation and disintegration, disabling their infectivity. In contrast to eukaryotic cells [61]–[65], direct DNA damage by the plasma-derived ROS and RNS cannot be excluded as a mechanism as the protective cytosol and multiple cellular membranes are missing in viruses. Obviously, programmed cell death programs and redox signaling events also will not be triggered in viruses, as seen in plasma-treated eukaryotic cells [66]–[69].

## IV. Gas Plasma Technology as Asset to Healthcare During Viral Pandemics Such as the SARS-COVID-19 Crisis

### A. Laboratory Assays for Coronavirus Research

The assessment of the efficacy of gas plasma technology for the inactivation of aerosol-transmitted viruses, such as coronavirus (CoV), relies on the use of laboratory assays for the detection of virus infectivity. Infection of the host by viruses represent a complex process, involving several actions (i.e., attachment, penetration, uncoating, replication, assembly, and release of new viral particles), each one requiring functional integrity of specific viral structures and the virus as a whole. In particular, the functionality of attachment proteins, the integrity of viral capsid, and the absence of significant damage to the viral genome are all required to accomplish the infection of the host cell. As all of these viral structures could be singularly or simultaneously affected by gas plasma treatments, complex *in vitro* systems as immortalized cell lines, that allow reproducing all stages of the infection cycle, are required to evaluate viral infectivity. This need, *per se*, represents a limitation to testing, as cell culture systems require dedicated laboratory environments and highly trained personnel to be efficiently implemented.

Further to this, several issues should be fully considered in relation to viral testing associated with gas plasma technology.

1) *Availability of Cell Culture Systems:* While several viruses of clinical interest can be readily cultivated in cell lines, some highly relevant human viral pathogens, as for example, norovirus and sapovirus, are not yet efficiently culturable.
2) *Biosafety Levels (BSLs) of the Viruses of Interest:* Diagnostic specimens containing highly pathogenic respiratory CoV, such as SARS, MERS, and SARS-CoV-2, can be manipulated in BSL-2 laboratories with additional personnel protective equipment (including but not limited to disposable gloves, gowns with cuffed sleeves, eye protection, full-face shields, strengthened disinfection procedures, etc.). However, for the propagation of these viruses in cell cultures (Vero E6 cells), BSL-3 facilities and work practices are strictly required (see WHO biosafety guidelines for handling of SARS specimens as of April 25, 2003; Interim Laboratory Biosafety Guidelines for Handling and Processing Specimens Associated with the Middle East Respiratory Syndrome Coronavirus (MERS-CoV)—Version 2; and Laboratory biosafety guidance related to coronavirus disease 2019 (COVID-19)—Interim guidance as of February 12, 2020).

The lack of *in vitro* cultivation systems or the limitations due to biosafety often prompts the use in experimental settings of surrogate viruses, i.e., viruses that are generally expected to mimic the behavior of the viruses they represent, without posing, however, the same technical and analytical problems. As far as CoV is concerned, given the structural and genetic similarity (capsid size, presence of envelope, and ssRNA genome), other human pathogenic CoV, usually responsible for mild to moderate respiratory illnesses (i.e., the common cold), might be used as surrogates for highly pathogenic CoV. These viruses include the alpha-CoV types 229E and NL63 and the beta-CoV types OC43 and HKU1, which are culturable in BLS-2 environments using, for example, MRC-5, LLC-MK2, HCT-8, and HAE cell cultures, respectively [70], [71]. It should be considered, however, that despite the lower pathogenicity of these strains, an assessment of the risk associated with potential exposure of laboratory personnel to these respiratory viruses should be undertaken before their selection for any experimental plan.

Considering these restrictions, animal CoV has been often proposed as surrogates for SARS and MERS. The swine and feline alpha-CoV transmissible gastroenteritis virus (TGEV) and feline infectious peritonitis virus (FIPV), for example, have been repeatedly used a surrogate to assess the recovery of CoV from water matrices and air conditioning/ventilation systems [72], [73], the virucidal effect of different chemical treatments [74], [75], and CoV survival in water or on surfaces [76]–[78]. Similarly, beta-CoVs as the bovine CoV and mouse hepatitis virus (MHV) have been used in studies on germicides [75], [79], for analytical methods validation [80], [81] and in persistence studies [76], [77]. More recently, to overcame biosafety issues, the use of CoV pseudoviral particles (i.e., defective viruses obtained by assembling relevant viral features onto a different viral backbone structure) has been proposed for the study of SARS-CoV-2 infectivity mechanisms [82], [83] suggesting—for the future—the possible application of *fusion* viruses also in other fields requiring the use of surrogates.

Although the use of human/animal CoV surrogates ensures high similarity of viral structures and therefore supports the inference of results to the viruses of interest, the concept of representativeness of surrogate viruses has been challenged [84], and it has been pointed out that surrogate coronaviruses responsible for gastrointestinal or hepatic diseases in animals may display a different resistance behavior in the environment and to treatments compared to human respiratory coronaviruses [85]. Further to this, the use of human/animal CoV surrogates does not overcome most of the feasibility issues associated with the complexity of cell culture systems. In fact, besides requiring dedicated environments and specialized personnel, cell culture systems suffer greatly from issues related to the experimental design and, particularly, to the matrix used for testing. While matrices as viral suspensions subjected to gas plasma treatments can easily undergo direct testing in cell cultures, recovery of viruses attached to solid surfaces (either inert or organic, as in the case of plasma studies on foods) poses several problems, including how to: 1) achieve an appropriate recovery efficiency to guarantee the reliability of results; 2) minimize damages to the viral structure to avoid overestimation of the inactivation by the process under testing; and 3) accomplish an adequate removal of all matrix residues to reduce interference and toxic effects on cell lines.

In order to prevent these problems and to reduce the technical complexity of viral testing on cell cultures, alternative strategies have often been proposed, mainly through the use of bacterial viruses (phages), whose detection/enumeration applies conventional bacteriological techniques and is less affected by the presence of matrix residues in the tested portion. As such, different phages have been used as a surrogate for CoV or, more in general, for enveloped viruses, with several studies, including either Enterobacteria phage MS2 (Leviviridae family) or Pseudomonas *phi* phages (es. $\phi 6$ or $\phi 12$, family Cystoviridae) [80], [86], [87]. It should be noticed, however, that representativeness of phages behavior for aerosol-transmitted viruses seems variable [88] and that neither of these surrogates displays full correspondence with CoV (MS2 phages being a nonenveloped virus and *phi* phages having a segmented double-stranded RNA). Other approaches to the by-pass cell culture of viruses include the use of the “viability PCR,” i.e., molecular assays specifically designated to assess the integrity of viral capsid or genome (or, more rarely, both). These approaches rely on specific analytical strategies as the use of long-template PCR, covering a large fragment or ideally the whole viral genome to assess its integrity, or the use of nucleic acids intercalating agents, as ethidium monoazide or propidium monoazide (EMA or PMA), to block the PCR amplification of virions in which the loss of capsid integrity has led to the exposure of viral RNA. Some of these strategies have also been applied in association with gas plasma treatment [89]. It should be considered, however, that the integrity of viral capsid and genome represent only a tile in the process of host cell infection and that, therefore, viability PCR methods can only approximate an assessment of viral infectivity.

### B. Coronavirus Decontamination of Air

In the last few years, including the period of recent emergency from COVID19, some papers dealing with the role of bioaerosol transport for virus respiratory diseases and the controversy between airborne and droplet transmission might be usefully mentioned. In particular, Bourouiba *et al.* [90] have taken into account the role of violent respiratory events (coughs and sneezes) in transferring respiratory diseases when infectious and susceptible individuals are in close proximity. Tangling experimental (high-speed imaging techniques) and theoretical investigations, the study draws attention to the importance of multiphase turbulent buoyant clouds with suspended droplets in potentially extending the range of respiratory pathogens to be taken into account. High sensitive laser light scattering observations confirmed that in confined environments normal speaking might cause airborne virus transmission, even from asymptomatic carriers of SARS-CoV-2. At the same time, loud speech induces the emission of thousands of oral fluid droplets per second [91]. The importance of airborne versus droplet routes needs to be highlighted, relevant for the transmission of many respiratory viruses and the consequent necessities of airborne precautions to be set in place in some cases [92]. In this regard, van Doremalen *et al.* [20] compared the transmission of SARS-CoV-2 to that of SARS-CoV-1 under different experimental conditions that include airborne and fomite (on plastic, stainless steel, cardboard, and copper substrates). They showed that viruses remain infectious in aerosols for many hours and on surfaces up to several days. This could explain nosocomial spread and superspreading while showing useful roadmap for efforts aimed at the mitigation of pandemics. A plasma-based alternative to protect against aerosolized pathogenic viruses would be a protective mouth-and-nose mask equipped with a battery-driven miniature plasma source.

### C. Concluding Assessment of the Opportunities for Decontamination of Coronavirus-Contaminated Body Surfaces

There is direct evidence that the probability of infection increases with exposure to viruses, and indirect evidence that the initial viral load influences the severity of the infection [93], [94]. To decrease the virus load and following the release of the infectious aerosol before diagnostic and therapeutic interventions in the upper respiratory tract (e.g., rhinoscopy, bronchoscopy, and bronchial lavage), as well as before dental treatment, the application of gas plasma treatment in the nasopharyngeal cavity could be effective. According to the current state of knowledge, the infectivity of SARS-CoV-2 is particularly severe because this virus replicates above all in the mouth and throat region. In an early stage of infection, symptoms of COVID-19 disease are not evident or still very truncated, while the infectivity of such individuals is particularly high [95]. Consequently, early reduction of viral loads in the mouth and throat region of positive tested but not yet sickened individuals likely reduces their infectivity and subsequently decreases or even abrogates further virus dissemination, while simultaneously reducing or even avoiding the drastic economic consequences of contact limitation and isolation. However, effective drug therapy for SARS-CoV-2 infection is not yet available. Alternatively, local treatment of infected mucosa might be taken into consideration. For this purpose, also processes based on gas plasma technology are discussed. It is well known that plasma treatment of air is useful for pollution control, including the reduction of airborne pathogens [96]–[101]. Moreover, plasma-treated gas or plasma-treated air, respectively, was proven to be highly effective in microbial decontamination of materials, surfaces, and goods [102]–[105]. Based on these facts, the idea was created to use plasma-treated air to reduce or eliminate the viral loads in the oral and pharyngeal cavities of intubated, ventilated patients. It was also proposed that gas plasma-mediated oxidation of cysteine could be one strategy for the alteration of SARS-CoV-2 pathogenicity, supplied potentially even via anesthetic masks during surgery [106]. However, and albeit this idea seems to be self-evident, some crucial boundary conditions cannot be neglected. First, mucosal tissue in the mouth and throat region is not a simple inanimate surface but a sensitive living tissue. Therefore, any mucosal tissue compatibility has to be proven beforehand in order to avoid severe local impairments and side effects. Possibly, this might not be the main problem because a recent study demonstrated that a direct gas plasma treatment of mucosal tissue in mice using the kINPen plasma jet was well tolerated and without side effects [107]. More important is the risk estimation of (at least partial) inhalation of plasma-treated air during the treatment of the mouth and throat region. It is well known that plasma treatment of nitrogen (N_2_) and oxygen (O_2_) containing air results in the generation of ozone (O_3_) and nitrogen oxides (NO_*x*_). Both gas species are essential for pathogen inactivation on the one hand but are highly toxic to the lungs on the other hand, if accumulating in higher concentrations. Therefore, it might be a challenge to balance the concentration of these gas species to achieve a sufficient antiviral effectivity while minimizing any side effects. Consequently, it has to be demanded that all approaches aiming at using plasma-treated breathing air for the inactivation of viruses in the mouth and throat region must guarantee and prove that any inhalation of this air is toxicologically acceptable.

Another field repeatedly under discussion is plasma application is hand disinfection [32], [108], [109]. It is not surprising that this approach is initiated again at some plasma institutes concerning SARS-CoV-2 reduction. As stated above, any conclusions by analogy from plasma effectivity to treat inanimate surfaces to the treatment of living skin have to be interpreted with caution. Besides proven skin tolerability above all, the plasma effectivity with regard to the strong requirements for hand and skin disinfection methods has to be demonstrated.

In general, any evidence of specific plasma effectivity against SARS-CoV-2 in living tissues like the skin or mucosa has not yet been provided.
